# Comparison of Total Variation with a Motion Estimation Based Compressed Sensing Approach for Self-Gated Cardiac Cine MRI in Small Animal Studies

**DOI:** 10.1371/journal.pone.0110594

**Published:** 2014-10-28

**Authors:** Juan F. P. J. Abascal, Paula Montesinos, Eugenio Marinetto, Javier Pascau, Manuel Desco

**Affiliations:** 1 Departamento de Bioingeniería e Ingeniería Aeroespacial, Universidad Carlos III de Madrid, Madrid, Spain; 2 Instituto de Investigación Sanitaria Gregorio Marañón (IiSGM), Madrid, Spain; 3 Centro de Investigación en Red de Salud Mental (CIBERSAM), Madrid, Spain; University of Maryland, College Park, United States of America

## Abstract

**Purpose:**

Compressed sensing (CS) has been widely applied to prospective cardiac cine MRI. The aim of this work is to study the benefits obtained by including motion estimation in the CS framework for small-animal retrospective cardiac cine.

**Methods:**

We propose a novel B-spline-based compressed sensing method (SPLICS) that includes motion estimation and generalizes previous spatiotemporal total variation (ST-TV) methods by taking into account motion between frames. In addition, we assess the effect of an optimum weighting between spatial and temporal sparsity to further improve results. Both methods were implemented using the efficient Split Bregman methodology and were evaluated on rat data comparing animals with myocardial infarction with controls for several acceleration factors.

**Results:**

ST-TV with optimum selection of the weighting sparsity parameter led to results similar to those of SPLICS; ST-TV with large relative temporal sparsity led to temporal blurring effects. However, SPLICS always properly corrected temporal blurring, independently of the weighting parameter. At acceleration factors of 15, SPLICS did not distort temporal intensity information but led to some artefacts and slight over-smoothing. At an acceleration factor of 7, images were reconstructed without significant loss of quality.

**Conclusion:**

We have validated SPLICS for retrospective cardiac cine in small animal, achieving high acceleration factors. In addition, we have shown that motion modelling may not be essential for retrospective cine and that similar results can be obtained by using ST-TV provided that an optimum selection of the spatiotemporal sparsity weighting parameter is performed.

## Introduction

Cardiac cine magnetic resonance imaging (MRI) has proved very useful for assessing heart motility and cardiac function. Cine imaging involves an intrinsic tradeoff between the acquisition time, spatiotemporal resolution, and signal-to-noise ratio (SNR) of the reconstructed images. Therefore, this application greatly benefits from acceleration techniques that reduce acquisition time. Traditional acceleration techniques were based on parallel imaging [1], [2]. The next generation of accelerating techniques—UNFOLD [3], k-t BLAST, and k-t SENSE [4]—exploited temporal redundancy. In the last few years, compressed sensing has achieved even higher acceleration factors [5]–[11].

Compressed sensing generates accurate image reconstructions from highly undersampled data obtained using randomized sampling and a nonlinear reconstruction algorithm that forces the image to be sparse in a transformed domain. Among the possible transform domains, one of the most widely used is the image gradient domain that leads to the minimization of the so-called total variation (TV). Reconstruction involves the solution of a convex constrained optimization problem, which can be accurately solved with classic optimization methods [12]. However, these are generally computationally expensive, and a common strategy is to approximate the problem by adopting an equivalent unconstrained optimization formulation. A new approach based on the Split Bregman method can accurately and efficiently resolve constrained optimization problems including L1-norm and TV penalty functions [13]–[15]. Split Bregman has been validated with static MRI [14] and cardiac cine data, in which the spatiotemporal TV (ST-TV) method is applied [16].

Motion-based reconstruction methods have been proposed as a generalization of temporal TV methods [17]–[19]. While temporal TV imposes sparsity by ensuring that few pixels change over time, a sparser transform can be achieved by incorporating knowledge of the motion across frames into the reconstruction algorithm. The above mentioned studies [17], [18], [19] focused on prospective cardiac cine and proved to be superior to previously used methods, such as kt-FOCUSS for human data [17] and to ST-TV for small-animal data [19].

Prospective cardiac cine requires the use of ECG recording and detection of R waves, which may be challenging in small animals at high field strengths, especially in the presence of pathologies such as myocardium infarction. This can be overcome by using retrospective cine, which relies on navigators to classify the data into different time frames [16], [20].

Substantial differences might be found in the behaviour of motion-based CS algorithms between its use in prospective human data and retrospective data applied to small animal. As opposite to humans, in the case of small animal, a higher heartbeat rate, lower SNR, different number of frames, and higher presence of artefacts motivate the repetition and averaging of several experiments. The repetition and averaging of data can be used to increase the acceleration achievable with CS, as the potential of CS depends on the level of randomization of the undersampling, which can be increased with randomization across repetitions in addition to the more usual encoding direction and temporal dimension [16].

All the aforementioned differences between prospective human data and retrospective animal data may influence the performance of CS methodologies. The aim of this work is to study the benefit of incorporating motion estimation within the compressed sensing framework into retrospective cardiac cine MRI for small-animal studies. To this purpose, we propose a novel B-spline-based compressed sensing method (SPLICS) based on motion estimation, which is evaluated by comparing with ST-TV using retrospective cardiac cine data from both healthy and infarcted rats. In addition, we assess the effect of an optimum weighting between spatial and temporal sparsity to further improve results with both SPLICS and ST-TV.

## Methods

### Compressed sensing

Compressed sensing enables accurate image reconstructions from randomly undersampled data [21], [22] by using convex optimization and imposing sparsity of the image in a transform domain, subject to a data fidelity constraint. Sparsity can be enforced by minimizing the L1-norm of the solution in the transform domain. With *f* as the measured k-space, *F* the undersampled Fourier operator, and *u* the image assumed to be sparse under the transform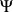, compressed sensing formulates the following constrained optimization problem
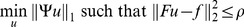
(1)


where ρ depends on the variance in signal noise as ρ  =  Nσ^2^, being N is the number of data points, for independent and identically distributed noise.

For static image reconstruction, the transformed domain chosen is usually the spatial gradient, 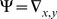, such that 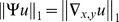 is the spatial TV.

### Compressed sensing for cardiac cine MRI

Dynamic cardiac applications are generally sparser in the temporal domain than in the spatial domain. In this work, we examine the effect of a spatiotemporal weighting parameter, α, that allows us to control the amount of sparsity enforced in one domain with respect to the other (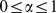). With this approach, a general compressed sensing reconstruction method that combines spatial and temporal sparsity can be written as
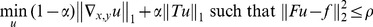
(2)


where both the image and raw data are represented by column vectors that comprise all frames, such that *u*  =  [*u*_1_^T^,…, *u*_I_^T^]^ T^, *f*  =  [*f*_1_^T^,…, *f*_I_^T^]^ T^, *I* is the number of cardiac phases, *F* and 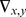 are operators that act frame by frame, and *T* is a sparsifying operator along the temporal dimension. Hereafter, we use isotropic spatial TV for the spatial sparsity transform 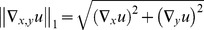.

#### ST-TV reconstruction

Spatiotemporal-total variation imposes sparsity on both spatial and temporal gradient domains [16]. As temporal gradient ensures that few pixels change over time, it is suitable for dynamic processes in which the background is static. By letting 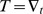 in equation (2), ST-TV becomes
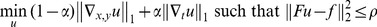
(3)


where the temporal gradient 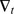 relates a pixel in a frame to the same pixel in the next frame, assuming a cyclic condition.

#### A compressed sensing approach using B-spline motion estimation (SPLICS)

**Figure 1:**
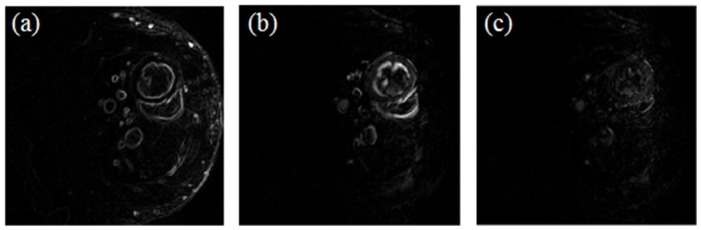
Full data reconstruction for end-diastole represented on the different transform domains. Sparsity of full data reconstruction on (a) spatial-gradient domain, |∇_xy_u_1_|, (b) temporal-gradient domain, |∇_t_u_1_|, and (c) motion-corrected domain, |Tu_1_|. Images are shown with the same window/level.

Motion-based reconstruction methods are based on simultaneous or alternating reconstruction of both motion and images from the data. If *T* in equation (2) represents an operator that encodes the motion between frames, thus relating pixels across frames, the compressed sensing problem for joint estimation of motion and images can be written as 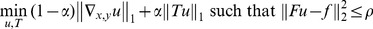
(4)


where both images *u* and the encoded motion *T* between frames are unknown. Modelling the motion across frames can be considered an extension of ST-TV that leads to a much sparser representation in the temporal domain. This is shown in figure 1 where full data reconstruction is represented on spatial gradient domain, 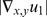, temporal-gradient domain, 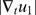, and motion-corrected domain, |Tu_1_|. Thus, in the case of a sparser temporal representation, one could increase the weight α enforcing temporal sparsity over spatial sparsity.

In our case, motion was estimated using a free-form deformation (FFD) model, which represents the motion in terms of cubic B-spline bases in order to preserve smoothness [23], [24]. B-splines have interesting properties such as positivity, symmetry, compact support and maximal order of approximation [25]–[27]. These properties make B-splines to be computationally efficient during interpolation and differentiation. FFD uses a sparse mesh of control points to model motion, which is interpolated using spline functions. A hierarchical approach enables the mesh of control points to be increased using multiresolution.

FFD has been widely used in medical image-registration to model deformation. The nonrigid registration problem searches for the spatial transformation R_i_:(x,y,z)→(x’,y’,z’), which maps any point from the image *u_i_* at the *i*th-frame to a point on the image at the following frame *u_i+_*_1_. Interpolation is then applied to relate pixel intensity between frames, with the result that *R_i_*(u*_i_*) comprises both registration and interpolation. The registration problem can be written as
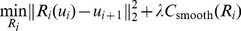
(5)


where *C*_smooth_ is a smoothness penalty function, generally in the form of a thin plate of metal bending energy [24]. For the implementation of the FFD-based registration method, we used the software available in MATLAB Central (Dirk-Jan Kroon; B-spline grid, image and point based registration; 2012, retrieved from <http://www.mathworks.com/matlabcentral/fileexchange/20057-b-spline-grid-image-and-point-based-registration>), which is based on the Quasi Newton L-BFGS optimization method. This approach requires selection of a threshold for the stopping criterion, the number of grid points, and the number of refinement grids within the hierarchical approach.

Given the registered images, the temporal sparsity transformation is obtained as 
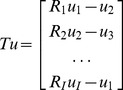
(6)


With regard to implementation of the SPLICS method, instead of solving equation (4), we propose an alternating two-step approach that estimates the motion in a first step and reconstructs the images using the estimated motion in a second step (pseudo-code is shown in Table 1). For the first estimate, u_estimate_, we used the solution given by ST-TV, which is only used to estimate the motion. It is important to remember that both registration and reconstruction steps are iterative processes. For the registration step, we used the nonrigid registration method from equation (5); for the reconstruction step, we used the Split Bregman method, as shown in the next section.

**Table 1:** Pseudo-code for the SPLICS method.

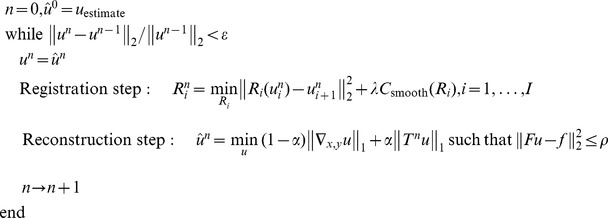

#### Implementation of ST-TV and SPLICS based on the Split Bregman formulation

We implemented ST-TV (equation (3)) and SPLICS (Table 1) using the Split Bregman method, which is computationally efficient for solving constrained optimization problems with L1-norm functionals [14]. The Split Bregman formulation separates L2- and L1-norm functionals in such a way that they can be solved analytically in two alternating steps. Constraints are imposed using the Bregman iteration as explained below. As motion-based reconstruction can be written as a generalization of the ST-TV, we develop the formulation for the general case and then provide details for an efficient computation in both ST-TV and SPLICS.

For solving equation (2) for a general operator *T* we generalize the Split Bregman formulation presented in [14], [16]. To allow for splitting, we include new variables, *d_x_*, *d_y_*, and *w*, and formulate a new problem equivalent to equation (2), as follows:
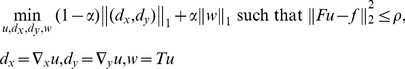
(7)


Using the Bregman iteration, this problem can now be easily handled by applying the equivalent unconstrained optimization problem and imposing constraints by adding a Bregman iteration *b_i_* for each constraint. Thus, 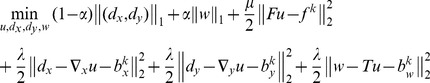
(8)


where *k* is the iteration number and the Bregman iterations are given by
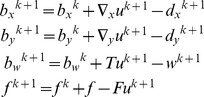
(9)


The Bregman iterations impose the constraints iteratively by adding the error back into the unconstrained formulation given in equation (8), thus making its solution converge to the solution of the constrained problem in equation (2). The last line in equation (9) enforces the data constraint, producing a sequence of solutions such that the solution error norm and the data fidelity term decrease monotonically. This framework is more robust than equivalent approximated unconstrained problems or continuation methods that impose constraints iteratively by slowly increasing the regularization parameters (for more details see [14]).

Note that *u* and the auxiliary variables are independent of each other; therefore, (8) can be split into several equations, one for each variable, and solved sequentially 
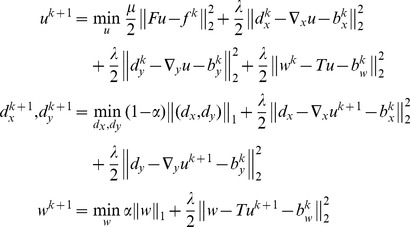
(10)


Since the solution of *u* only involves L2-norm functionals, it can be found exactly by differentiating the cost function and equating it to zero. The resulting linear system, which corresponds to a Gauss-Newton step, is given by 
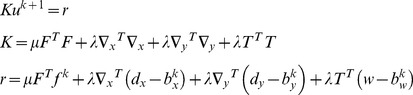
(11)


Note that the solution *u* is obtained analytically from a quadratic penalty function; therefore, an estimation of the step-size is not required. The linear system constitutes a very large scale problem, where *K* = N^2^×N^2^ (N = 192), yet it can be efficiently solved using a Krylov solver, which involves only matrix-vector multiplications, as follows:
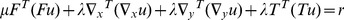
(12)


Here, we used the biconjugate gradient stabilized as the Krylov solver. This methodology has been applied in SPLICS, where *F*, 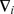, and *T* can be treated as general operators.

For ST-TV, all operators,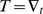, *F*, 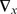, 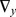, and 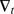, and the variables, *u*, *d*, and *w*, can be expressed in the Fourier domain, with dimension N×N. In this case, solving equation (12) in the Fourier domain is much faster than the equivalent Krylov solver in the image domain. In the case of SPLICS *T* cannot be represented in the Fourier domain and thus the problem has to be solved in the image domain [14], [16].

For both ST-TV and SPLICS, *d_x_*, *d_y_*, and *w* are solved analytically using shrinkage formulas, which are thresholding operations [14], [28]

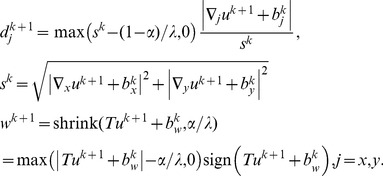
(13)


### Test data set and undersampling

#### Test data set

**Figure 2:**
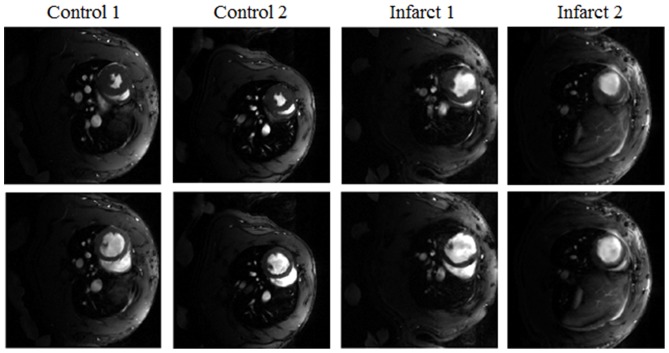
End-systole (first row) and end-diastole (second row) from fully-sampled data reconstruction.

Self-gated rat cardiac cine sequences, IntraGateFLASH, were acquired with a 7T Bruker Biospec 70/20 scanner using a linear coil resonator for transmission and a dedicated four-element cardiac phased array coil for reception. Acquisition parameters were: TE = 2.43 ms, TR = 8 ms, number of total repetitions = 200, number of frames = 8, matrix size = 192×192, FOV = 5×5 cm, slice thickness = 1.2 mm, and acquisition time = 5 min 7 s. Four Wistar male rats (weight 300 g to 350 g) were used in this study divided in healthy animals (wild type) and infarct animals (figure 2). Two out of four animals were induced with isoflurane (4%), intubated and place on isoflurane (2%) anaesthesia with mechanical ventilation for the duration of the surgical procedure. We induced a myocardial infarction by permanent left anterior descending artery occlusion as described previously [29]. The animals were exposed to 12/12 light/dark circle and allowed access to food and water ad libitum. Animals were handled according to the European Communities Council Directive (2010/63/UE) and national regulations (RD 53/2013) and under the approval of the Animal Experimentation Ethics Committee of Hospital General Universitario Gregorio Marañón.

#### Undersampling pattern

In order to reduce acquisition time, it is necessary to skip phase encoding lines in the acquisition scheme. One of the requirements of the compressed sensing technique is that the undersampling pattern should be random in order to produce incoherent artifacts [22]. By adapting the methodology proposed by Lustig *et al*. [22], in our work, we create quasi-random undersampling patterns in which randomization is performed in the phase encoding direction and through the temporal dimension.

**Figure 3:**
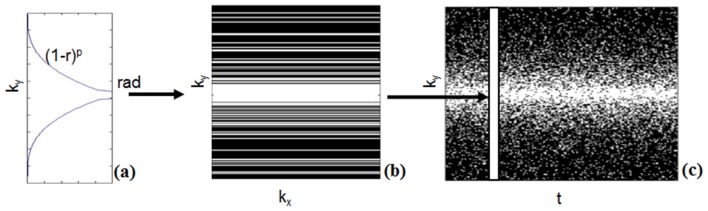
Undersampling pattern strategy used in this study in which randomization is performed along both phase encoding and temporal directions. Probability density function in (a) is used to generate undersampling patterns that are random in the phase encoding direction (b). Time randomization is achieved by changing the undersampling pattern for each temporal instant (c).

A polynomial probability density function (figure 3(a)) that assigns sampling probabilities to different regions of k-space was used to generate undersampling patterns that are random in the phase encoding direction (figure 3(b)). Then, changing the undersampling pattern for each time point leads to an undersampling acquisition scheme that is random in both phase encoding and temporal directions (figure 3(c)).

Undersampling patterns were simulated using the fully sampled dataset. After the undersampling process, each of the remaining phase encoding lines is binned into a specific cardiac frame (8 final frames), where coincident phase encoding lines are averaged. This averaging step, in combination with randomization over time, results in that the percentage of non-zero data in the final k-spaces is higher than the percentage of data actually acquired [16]. To avoid confusion, two different terms are defined: ‘acceleration factor’ (indicated with an x), which represents the speed up rate with respect to the fully sampled data, and ‘percentage of filled k-space’ (FS), which corresponds to the percentage of nonzero lines after the averaging step. For example, a given undersampling pattern can preserve only 7% of the acquired data, providing an acceleration factor of ×15; however, after the classification and averaging step, FS = 26%.

The reconstruction process was performed separately for each element of the phased array antenna and combined with a sum of squares operation.

### Evaluation

For an acceleration factor ×7 (FS = 60%) from the complete data (percentage of filled k-space shown within parenthesis), the effect of the spatiotemporal weighting parameter α was analyzed in the range 0.99 to 0.01 (α  = 0.99, 0.9, 0.7, 0.5, 0.3, 0.1, 0.01), for the data set 1. SPLICS and ST-TV results were compared to better understand the benefit of including motion estimation. The number of iterations (k in equation (10)) had to be selected for both ST-TV and SPLICS. To ensure the best result for each method, we chose the iteration numbers that provided minimum solution error, adopting the full data reconstruction (figure 2) as the gold standard.

We analyzed the results obtained by both methods for acceleration factors ×7 (FS = 60%), ×10 (FS = 40%), ×15 (FS = 26%), ×20 (FS = 22%) for all data sets.

Reconstructed images were evaluated in terms of the relative solution error norm within a region-of-interest (ROI) that comprised the whole heart. The presence of artifacts in reconstructed images was analyzed by visual inspection, and temporal blurring was evaluated by measuring temporal intensity changes on a circular ROI located in the inner part of the myocardium (diameter = 6 pixels).

## Results

### Effect of the spatiotemporal weighting parameter

**Figure 4:**
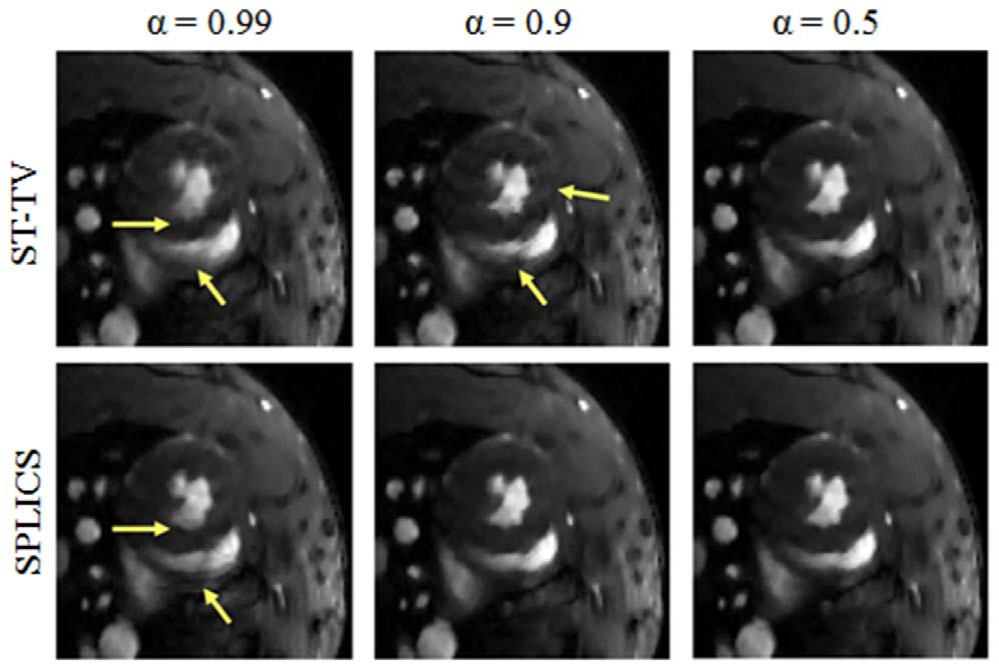
Image zoom corresponding to an acceleration factor ×7 (FS = 60%) reconstructed with ST-TV and SPLICS for three different values of the spatiotemporal weighting parameter α (0.99, 0.9, 0.5). The arrows indicate locations where temporal blurring and artifacts are more noticeable. All images have the same window/level.

We show reconstructed images for 3 different values of the spatiotemporal weighting parameter α (α = 0.99, 0.9, and 0.5) for ST-TV and SPLICS. The outcome of ST-TV proved to be similar to that of SPLICS for a wide range of the weighting parameter (α≤0.5) (figure 4). From α = 0.99 to α = 0.9 (large temporal weight), ST-TV led to significant temporal blurring effects and motion artifacts. SPLICS was more robust against the selection of α. SPLICS corrected for temporal blurring effects and motion artifacts for α = 0.9 and α = 0.5 and reduced them for α = 0.99. The best results were obtained with ST-TV for α = 0.5 and with SPLICS for α = 0.9 and α = 0.5, with no noticeable differences between these results. For values of α lower than 0.5, images remain similar to those obtained with α = 0.5 (images not shown).

**Figure 5:**
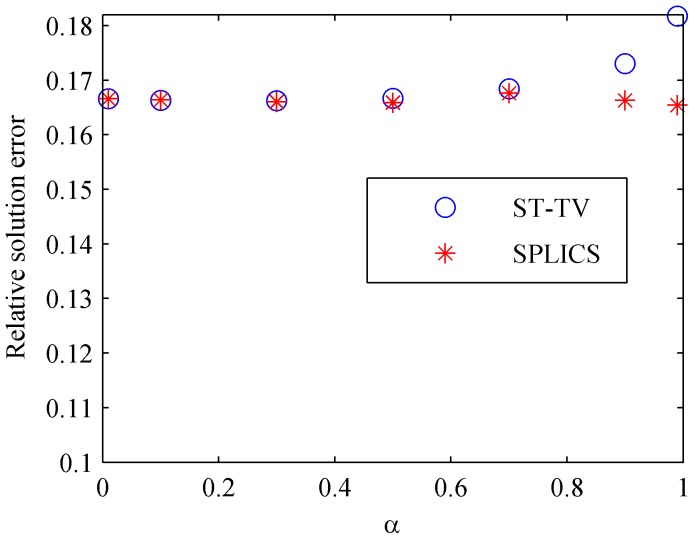
Relative solution error norm corresponding to an acceleration factor ×7 (FS = 60%) reconstructed with ST-TV and SPLICS (images in figure 4) for different values of the spatiotemporal weighting parameter α.

Visual findings are in agreement with the normalized solution error norm shown in figure 5. For ST-TV, α≤0.5 yielded the lowest solution error, and increasing α from 0.7 to 0.99 increased the solution error. On the other hand, SPLICS maintained low solution error for all values of α.

**Figure 6:**
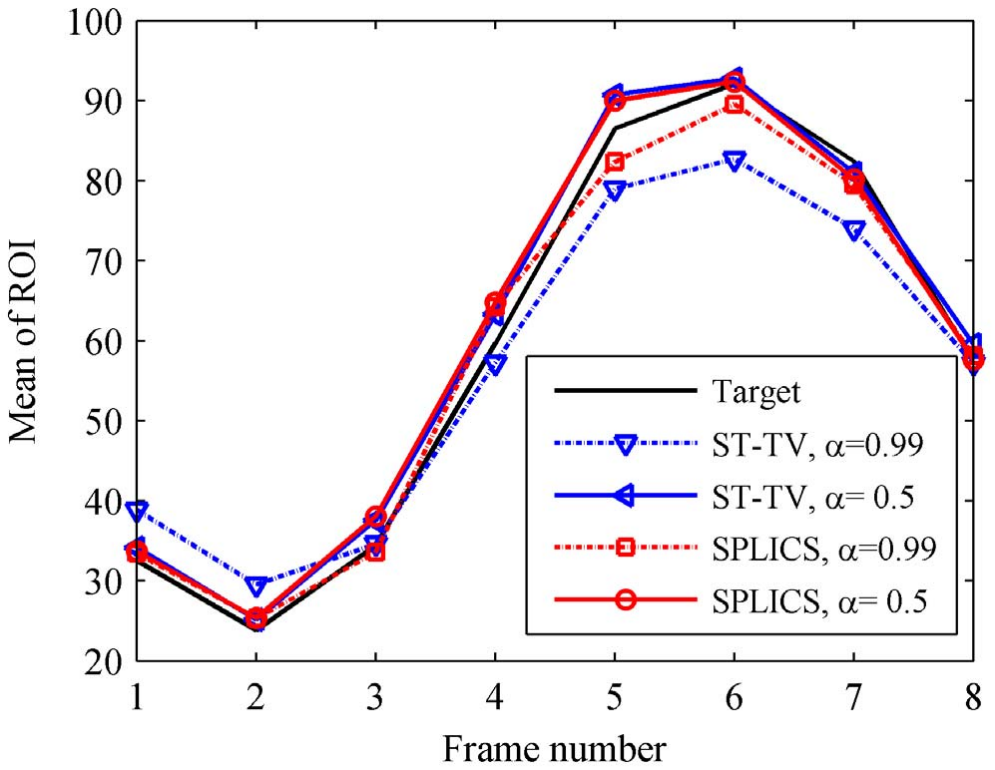
Temporal intensity changes, corresponding to a circular ROI located within the myocardium, for the images reconstructed with ST-TV and SPLICS (see also figure 4).

Figure 6 shows temporal intensity changes for ST-TV and SPLICS. For α = 0.5 both methods recovered the temporal data; for α = 0.99, ST-TV presented higher temporal blurring than SPLICS.

As for the reconstruction parameters, the initial image was estimated using ST-TV with parameters λ = 1 and μ = 2. For the registration step, we used three meshes of control points and a hierarchical approach (8×8, 12×12, and 20×20), with a convergence tolerance of 10^−4^. For the reconstruction step, we empirically chose λ = 1 and μ = 2. We applied only one iteration of the alternating method (*n* = 1 in Table 1), as further iterations did not significantly improve these data.

### Analysis of acceleration factor achieved

**Figure 7:**
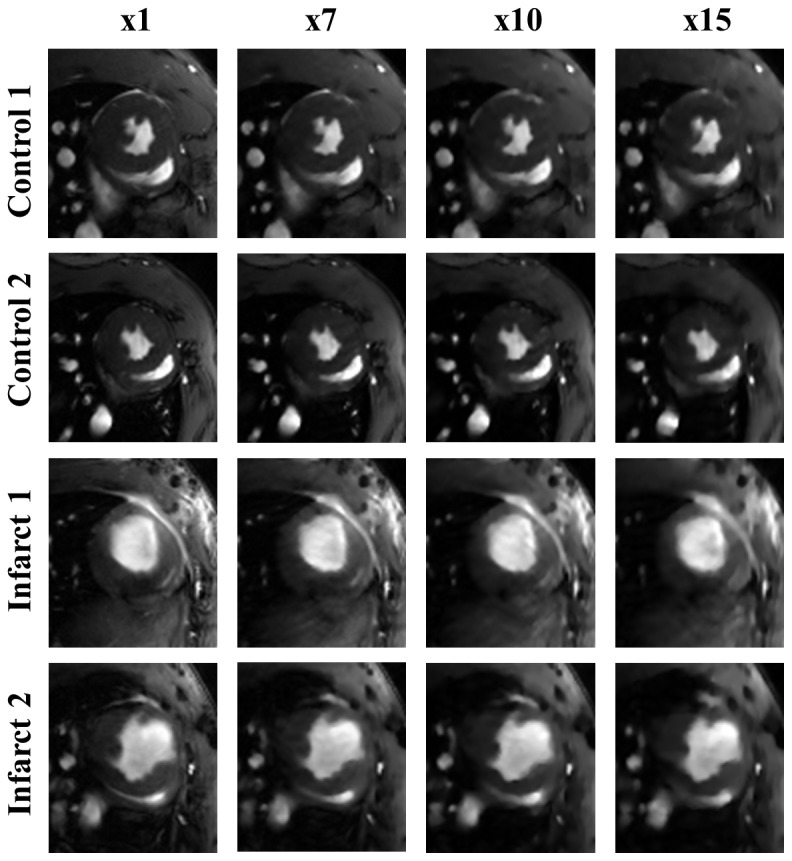
Image zoom of the end-systole corresponding to acceleration factors ×7 (FS = 60%), ×10 (FS = 40%) and ×15 (FS = 26%) reconstructed with SPLICS for a spatiotemporal weighting parameter α = 0.5. Images show different window/level for each subject, in order to make them look similar.

Figure 7 shows the end-systole reconstructed with SPLICS for acceleration factors ×7 (FS = 60%), ×10 (FS = 40%) and ×15 (FS = 26%), for α = 0.5 (due to the similarities between SPLICS and ST-TV, we only show results given by SPLICS). For acceleration factors up to ×7, image quality is close to that of the fully-sampled data. Decreasing the percentage of data acquired resulted in a slight increase in artefacts and in the smoothing effect from ×10 (FS = 40%) to ×15 (FS = 26%). For ×20 (FS = 22%), reconstructed images were strongly over-smoothed (results not shown).

**Figure 8:**
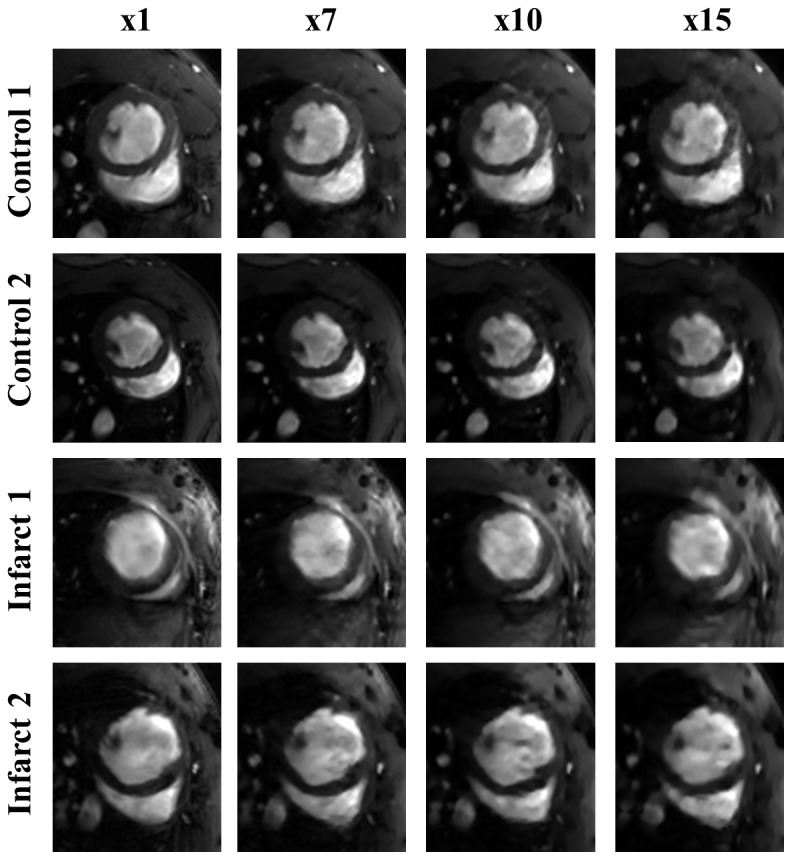
Image zoom of end-diastole corresponding to acceleration factors ×7 (FS = 60%), ×10 (FS = 40%) and ×15 (FS = 26%) reconstructed with SPLICS for a spatiotemporal weighting parameter α = 0.5. Images show different window/level for each subject, in order to make them look similar.

Figure 8 shows the end-diastole (equivalent to figure 7) reconstructed with SPLICS for acceleration factors ×7 (FS = 60%), ×10 (FS = 40%) and ×15 (FS = 26%). Like in systole, decreasing the percentage of acquired data results in an increase in artefacts together with some smoothing effect. However, the diastole is more prone to motion and flow artefacts than the systole. This effect is more clear in control subjects.

**Figure 9:**
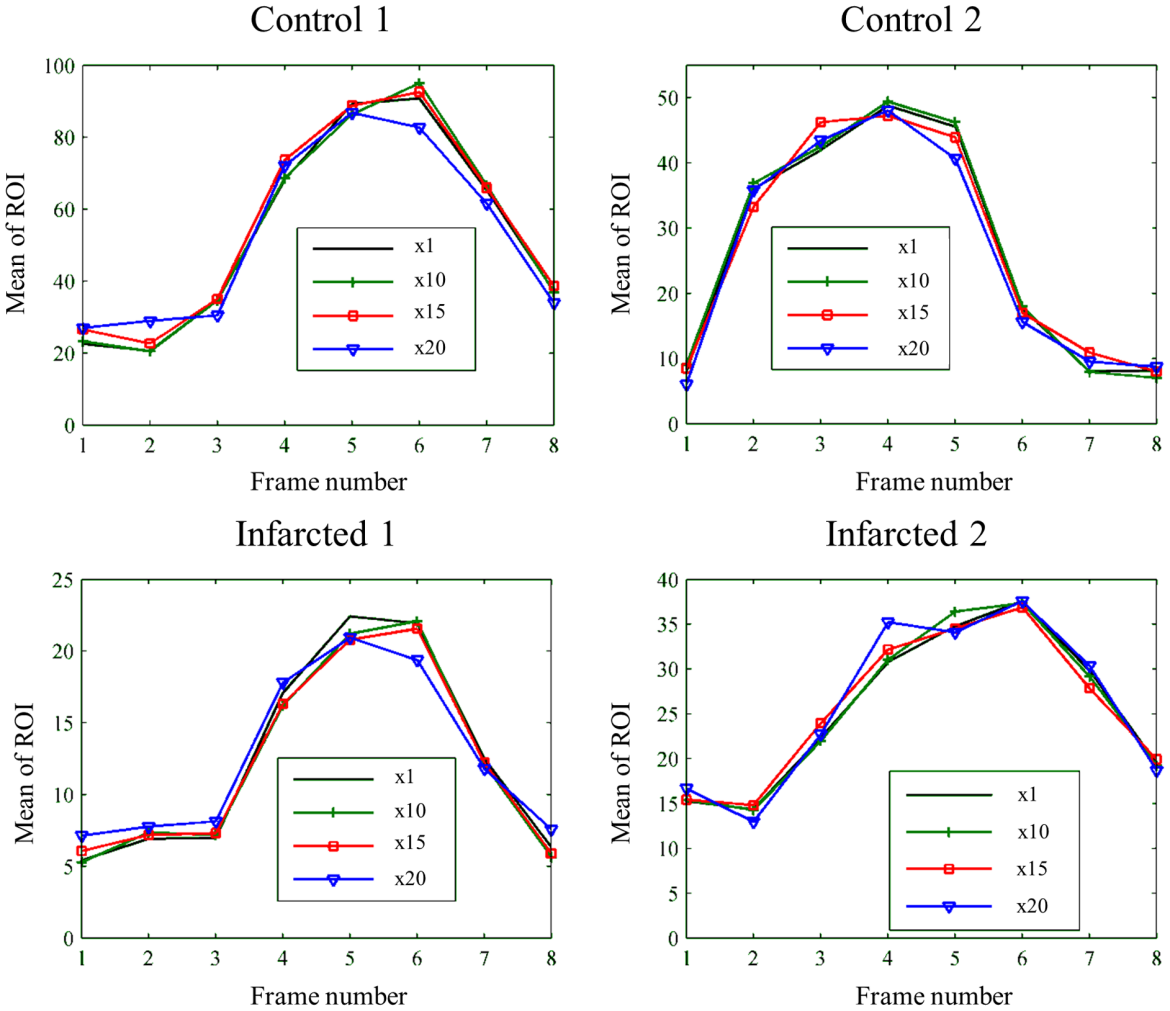
Temporal intensity changes for the images shown in figure 8 for all data sets, measured on a circular ROI (diameter = 6 pixels) located at the inner part of the myocardium.

Temporal intensity changes are shown in figure 9. For acceleration factors ×10 (FS = 40%) and ×15 (FS = 26%), temporal intensity changes are similar to those in the fully sampled data; for ×20 (FS = 22%), temporal smoothing becomes more significant.

Regarding convergence, the iteration number that provided best results varied in the range from 105 to 380 iterations for the different subjects and undersampling factors, with larger variation across subjects than across undersampling factors. As in a real compressed sensing acquisition the target image is not available, we tested the effect of reconstructing all data sets with a fixed number of iterations, comparing results given by 100, 200 and 300 iterations, and found images to be similar (data not shown).

Computation time for ST-TV was 0.12 s per iteration (Intel Core i7-3770 CPU 3.40 GHz, 8 GB RAM, 64-bit operating system), and it took 1.5 minutes to reconstruct all the images with 200 iterations (eight frames, four array elements). In each iteration, the heavier part of the computation was two 3D FFT and two 2D FFT image frame-by-image frame, which were required for inversion of the linear system in the Fourier domain. In the case of SPLICS, the registration step took 1.5 minutes and the computation time for the reconstruction step depended on the tolerance of the Krylov solver used for the inversion of the linear system in the image domain: 2.4 s per iteration for a tolerance of 10^−4^ and 1.2 s per iteration for a tolerance of 10^−2^. As the linear system does not need to be solved with high precision, a tolerance of 10^−2^ was sufficient and gave the same solution as 10^−4^. In this case, SPLICS took 15.5 minutes to reconstruct all the images with 200 iterations. A straightforward parallelization over the four phase arrays reduced computation times to 55.5 s for ST-TV and 6.6 minutes for SPLICS (35 seconds for the registration step and 6 minutes for the reconstruction step).

## Discussion

We studied the influence of incorporating motion estimation in the compressed sensing framework for retrospective cardiac MRI in small animal. To this end, we proposed a new motion-based reconstruction method (SPLICS) and compared it with ST-TV, as SPLICS can be considered as a generalization of ST-TV that also models motion. In addition, we investigated the effect of the spatiotemporal weighting parameter intended to control the relative degree of spatial and temporal sparsity.

Previous works have combined spatial and temporal sparsifying operators [16], [19], [30]. In our work we compared the effect of the spatiotemporal weighting parameter on both ST-TV and motion-based reconstruction. We found that ST-TV with low relative temporal weighting (α≤0.5) led to similar results than SPLICS. When temporal weighting was increased (α>0.5) ST-TV led to images with temporal blurring effects and motion artifacts on the moving parts of the image. On the other hand, SPLICS appeared more robust for all α values, as it also takes into account the motion information of the moving parts of the images. Hence, from our results it seems that for retrospective cardiac cine in small animal ST-TV with an optimum weighting parameter provides good results and does not require motion modelling.

A motion-based reconstruction method has been shown to improve k-t FOCUSS for prospective human cine [17] and ST-TV for prospective small-animal cine [19]. In [19] it was found that ST-TV led to temporal blurring while motion-based reconstruction corrected these effects. In contrast, for retrospective data we obtained that ST-TV with optimum selection of the spatiotemporal weighting parameter did not lead to temporal blurring artifacts, providing a solution similar to that of SPLICS. Thus, motion-based reconstruction would provide an improvement only in those cases where other methods lead to temporal blurring effects.

While motion-based reconstruction has led to better results than k-t FOCUSS and ST-TV for prospective data, we found similar results between SPLICS and ST-TV for retrospective data. These discrepancies between our results and previous ones may be due to differences in the acquisition process and in the way the undersampling process was applied. In our case, for retrospective small-animal cine, we have taken advantage of the repetition and averaging step by also randomizing the undersampling pattern across repetitions. With the prospective cine dataset the undersampling scheme led to a number of different undersampling patterns (figure 3(b)) equal to the number of frames. However, with the retrospective case the undersampling scheme used produced a number of different undersampling patterns equal to the number of repetitions (figure 3(c)), which is 17 times higher than the number of frames. In this way, the retrospective acquisition benefits from higher randomization. The same strategy could be also applied to prospective sequences by changing the undersampling pattern across different averages during the acquisition, but, to the best of our knowledge, no previous works have addressed this strategy for motion-based reconstruction. However, although randomization across repetitions can be applied to the prospective sequence, some differences still remain between prospective and retrospective undersampling processes. While the prospective acquisition scheme allows us to decide in advance which undersampling pattern will be applied to each frame, this is not possible for retrospective cine. In the retrospective case once the acquisition has finished, acquired phase-encoding lines are binned into cardiac frames based on the recorded navigator data, making it impossible to determine in advance the undersampling pattern on each frame [16].

The claimed acceleration factor depends on the quality criterion and the tolerance to artefacts. Adopting the criterion of preservation of image quality, for acceleration factors up to ×7 (FS = 60%), image quality is close to that of the fully-sampled data. Higher accelerations resulted in an increase of the smoothing artifact. However, adopting the criterion of temporal intensity changes in an ROI in the myocardium and the presence of artefacts at end-systole/diastole, which are the frames used to extract functional measurements such as ejection fraction and left-ventricle mass [31], results showed that accelerations up to ×10 (FS = 40%)−×15 (FS = 26%) are feasible. For an acceleration factor ×10 the important features in the image were preserved and images presented high resolution. An acceleration factor ×15 led to a slight increase in artefacts and over-smoothing effect, without distorting temporal intensity information. Higher accelerations led to over-smoothed images that affected both resolution and temporal intensity changes. However, we remark that image quality in both the fully sampled and undersampled data sets is variable across frames. End of systole and end of diastole showed good quality in all cases but some intermediate frames presented some motion artefacts that became more conspicuous as the acceleration increased. Comparing to previous works, a wide range of acceleration factors can be found in the literature. However, most works focused on prospective clinical applications and only two included retrospective reconstruction [6], [32]. Of the few studies addressing preclinical small-animal applications [16], [33]–[36], only two used retrospective self-gated cine sequences, obtaining acceleration factors of 3 [36] and 15 [16] with a ST-TV method similar to the one used in this paper.

To our knowledge, our work is the first one that studies motion estimation not only in normal cases [19] [17] but also in rats with myocardial infarction. We found small differences between infarcted and healthy animals. Temporal intensity changes were slightly better recovered in infarcted animals because the myocardium motion is compromised in this case. Motion artefacts were more apparent for healthy animals.

Previous work that proposed motion-based reconstruction methods modeled motion using optical flow methods [18], block-matching algorithms and phase-based motion estimation [37]. The latter was found to be more robust than block-matching algorithms and optical flow methods [17]. Our motion estimation step consisted of free-form deformation, which is a widely used nonrigid registration method in medical imaging, based on hierarchical B-splines [23], [38]. However, further work would be required to better understand the differences among motion estimation methods for cardiac cine compressed sensing MRI.

Our study is subject to a series of limitations. Our conclusions were drawn from an undersampled dataset made from fully sampled data instead of being directly acquired. Further work will focus on validation with real-time undersampled data and analysis of the effect of randomization across repetitions for prospective cine, which may further increase the potential of CS. In this work we focused on the comparison between SPLICS and ST-TV, assessing the quality of the reconstruction based on local image quality criteria. To determine acceleration factors achievable in real scenarios, a much larger dataset including both patients and control datasets and the evaluation of global metrics, such as ejection fraction, will be required. It is possible that a given method, even being better in technical terms, does not increase clinical usefulness, but this issue is out of the scope of our work.

## Conclusion

We studied the benefit of motion-based reconstruction for retrospective cardiac cine in small-animal studies, using SPLICS, a new motion-based reconstruction method. We also compared SPLICS to ST-TV and analyzed the effect of the spatiotemporal sparsity parameter. We found that ST-TV with optimum α leads to results similar to those of SPLICS. On the other hand, SPLICS is more robust for the selection of the weighting parameter and provided similar or superior results in all our cases. Hence, we have validated SPLICS and found that for retrospective cardiac cine ST-TV with optimum spatiotemporal weighting parameter is a good methodology for accelerating retrospective cardiac cine MRI in small animals.
